# A multicentre clinical validation of AminoIndex Cancer Screening (AICS)

**DOI:** 10.1038/s41598-019-50304-y

**Published:** 2019-09-25

**Authors:** Haruo Mikami, Osamu Kimura, Hiroshi Yamamoto, Shinya Kikuchi, Yohko Nakamura, Toshihiko Ando, Minoru Yamakado

**Affiliations:** 1 Chiba Cancer Center Research Institute, Chiba, Japan; 2 Hakuai Hospital, Tottori, Japan; 3 Ajinomoto Co. Inc., Kanagawa, Japan; 4 Department of Nursing, Ashikaga University, Tochigi, Japan; 5 Center for Multiphasic Health Testing and Services, Mitsui Memorial Hospital, Tokyo, Japan

**Keywords:** Computational biology and bioinformatics, Diagnostic markers

## Abstract

AminoIndex Cancer Screening (AICS) is a novel cancer screening test based on plasma free amino acid (PFAA) levels. This system categorises subjects as rank A, B, or C in order of increasing probability of each cancer incidence. The current study aimed to validate the potential of AICS for cancer detection. AICS values were determined from the PFAA levels in subjects examined at Chiba Cancer Center Cohort, Mitsui Memorial Hospital, and Saihaku Hospital, and the cancer incidence was investigated. The sensitivities of rank C for cancer diagnosis within 1 year after AICS examination were 83.3% (10/12) for gastric, 50.0% (2/4) for lung, 46.2% (6/13) for colorectal, 50.0% (8/16) for prostate, 43.8% (7/16) for breast, and 50.0% (1/2) for uterine/ovarian cancer. The total cancer detection rate via AICS was 0.33% (34/10,245). The sensitivities during the maximum follow-up period of 6.2 years were 51.7% (15/29) for gastric, 18.2% (2/11) for lung, 28.6% (8/28) for colorectal, 36.4% (8/22) for prostate, 29.0% (9/31) for breast, and 33.3% (2/6) for uterine/ovarian cancers. In conclusion, AICS is a more useful method for evaluating the probability of cancer incidence than for predicting onset, suggesting that annual AICS should be recommended to detect any malignancy.

## Introduction

Cancer mortality has been constantly increasing in Japan, and cancer has been the leading cause of death since 1981^1^. In 2016, 28.5% of Japanese people died of cancer, and this is attributed to prolonged life expectancies and changes in dietary habits. As such, the prevention and early detection and treatment of cancer are becoming increasingly important. Cancer screening is known to be among the most effective strategies for early detection of cancer. Currently, the Japanese government distributes vouchers and provides free consultation services to encourage their citizens to undergo cancer screening. Despite these efforts, one of the current problems of cancer screening in Japan is a low medical examination rate because medical examinations for individual cancer types are time consuming and cause a physical burden. Moreover, some screening tests have low accuracy. Thus, a more convenient screening method is expected to increase the examination rate.

Metabolomics is one of the approaches intensively investigated for cancer diagnosis^2–5^. Particularly, because amino acids play essential physiological roles both as basic metabolites and metabolic regulators, plasma free amino acids (PFAAs) are promising biomarkers. PFAA profiles are known to be influenced by metabolic variations induced by specific diseases such as various cancers, liver failure, renal failure, or inflammatory bowel disease^6–9^.

As for cancer screening, changes in PFAA profiles have been examined in various cancers and has led to the development of AminoIndex Cancer Screening (AICS) test. AICS was developed using the “AminoIndex Technology,” which scores the probability of disease occurrence via multivariate analysis with PFAA concentration as a variable^10,11^. Currently, AICS is used in clinical practice in Japan to screen for gastric cancer, lung cancer, colon cancer, pancreatic cancer, prostate cancer, breast cancer, and uterine/ovarian cancer^12–15^.

Specifically, AICS was developed based on a multivariate analysis of PFAA concentrations compared with approximately 2,900 patients with various cancer and approximately 13,000 healthy controls through a case-control study. AICS (gastric), AICS (lung), AICS (colorectal), AICS (pancreatic), AICS (prostate), and AICS (breast) are screening markers for the corresponding cancer. Because PFAA changes were similar in cervical, endometrial, and ovarian cancer, AICS(uterine/ovarian) was developed for comprehensive screening of these three cancer types. From the amino acid data, the probability of each cancer incidence is expressed as a numerical AICS value of 0.0 to 10.0. As a criterion of the probability of each cancer, rank A is ranged from 0.0 to 4.9, rank B is ranged from 5.0 to 7.9, and rank C is ranged from 8.0 to 10.0^16–20^. The higher AICS value, the higher the probability of each cancer incidence. In this study, we aimed to validate the clinical usefulness of AICS for cancer detection.

## Results

### Subject characteristics

**Figure 1:**
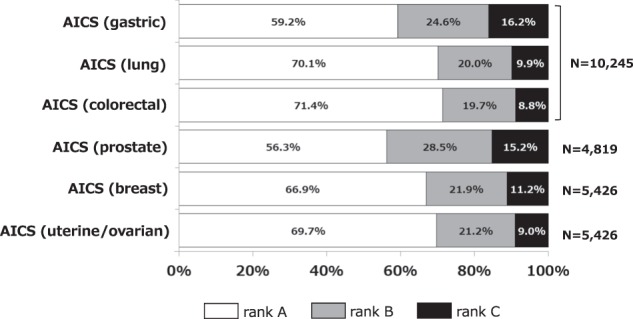
AICS rank distribution. White, grey and black parts show the distribution of rank A, B and C, respectively.

The cohort comprised 10, 245 individuals who underwent AICS in Chiba Cancer Center cohort (N = 2,886, research cohort spanning urban area to rural area), Mitsui Memorial Hospital (N = 4,967, medical check-up in urban area), and Saihaku Hospital (N = 2,392, medical check-up in rural area). The male-to-female ratio was 1: 1.1. The mean age ± standard deviation of all 10,245 subjects (4,819 men and 5,426 women) at the time of the blood sampling was 59 ± 11 (range 24–93) years. The distribution of subjects by sex and age in each facility are shown in Table 1. The rank distribution of each AICS is shown in Fig. 1. All 10,245 subjects were examined using AICS (gastric), AICS (lung), and AICS (colorectal), whereas 4,819 men were examined using AICS (prostate), and 5,426 women were examined using AICS (breast) and AICS (uterine/ovarian). In total, 16.2%, 9.9%, 8.8%, 15.2%, 11.2%, and 9.0% of patients were categorised as rank C in AICS (gastric), AICS (lung), AICS (colorectal), AICS (prostate), AICS (breast), and AICS (uterine/ovarian),respectively. In total, 127 patients were diagnosed with cancer within the follow-up period (gastric cancer, 29; lung cancer, 11; colorectal cancer, 28; prostate cancer, 22; breast cancer, 31; endometrial cancer, 4; and ovarian cancer, 2) (Table 2). Of these, approximately 50% (63 patients) were diagnosed within 1 year after AICS examination. The maximum follow-up period was 6.2 years. Regarding cancer patients, 83.3%, 50.5%, 46.2%, 50.2%, 43.8%, and 50.0% of the corresponding cancer patients were categorised as rank C in AICS (gastric), AICS (lung), AICS (colorectal), AICS (prostate), AICS (breast), and AICS (uterine/ovarian), respectively (Fig. S1).

**Table 1:** Subject characteristics.

	Chiba Cancer Center Cohort	Mitsui Hospital	Saihaku Hospital	Total
Number of subjects	2,886	4,967	2,392	10,245
Age (average ± SD)	58 ± 11	58 ± 11	63 ± 11	59 ± 11
Sex (Male/Female)	1,033/1,853	2,757/2,210	1,029/1,363	4,819/5,426
Age: 20–29 years	—	14/18	—	14/18
Age: 30–39 years	71/146	133/131	9/10	213/287
Age: 40–49 years	162/327	401/392	130/197	693/916
Age: 50–59 years	169/490	738/612	201/308	1,108/1,410
Age: 60–69 years	467/732	1,002/724	395/481	1,864/1,937
Age: 70–79 years	164/158	412/291	228/288	804/737
Age: 80–89 years	—	57/42	63/74	120/116
Age: 90 + years	—	—	3/5	3/5

**Table 2:** Number of subjects diagnosed with cancer at the different time periods.

Type of cancer		All cases	Within 1 year	After more than 1 year
Gastric cancer		29	12	17
Lung cancer		11	4	7
Colorectal cancer		28	13	15
Prostate cancer		22	16	6
Breast cancer		31	16	15
Uterine/ovarian cancer	Cervical cancer	0	0	0
	Endometrial cancer	4	1	3
	Ovarian cancer	2	1	1
Total		127	63	64

### Sensitivity

**Figure 2:**
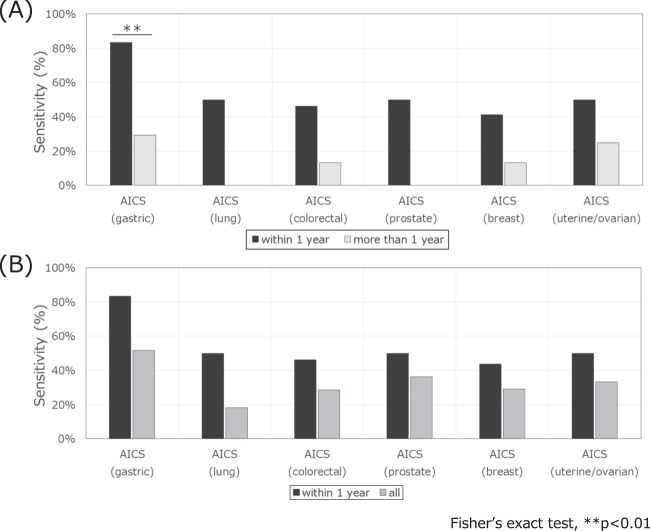
Sensitivity of AICS for each type of cancer in rank C. (**A**) Comparison of sensitivity within 1 year (black bars) and at more than 1 year (light grey bars) after AICS examination. (**B**) Comparison of sensitivity within 1 year (black bars) and all follow-up periods (dark grey bars) after AICS examination. Significant difference between groups (p < 0.01, Fisher’s exact test).

The sensitivities of the corresponding cancer in rank C within 1 year after AICS examination were 83.3% (10/12) for AICS (gastric), 50.0% (2/4) for AICS (lung), 46.2% (6/13) for AICS (colorectal), 50.0% (8/16) for AICS (prostate), 43.8% (7/16) for AICS (breast), and 50.0% (1/2) for AICS (uterine/ovarian). One patient who developed ovarian cancer was classified as rank C in AICS (uterine/ovarian). Meanwhile, the sensitivities of the corresponding cancer in rank C at more than 1 year after AICS examination were 29.4% (5/17) for AICS (gastric), 0% (0/7) for AICS (lung), 13.3% (2/15) for AICS (colorectal), 0% (0/6) for AICS (prostate), 13.3% (2/15) for AICS (breast), and 25.0% (1/4) for AICS (uterine/ovarian) (Fig. 2A, Table S1). The sensitivity of AICS (gastric) was significantly higher within 1 year than that at more than 1 year (Fisher exact test: P < 0.01). The sensitivities of AICS for other cancers were also higher within 1 year than that at more than 1 year, but the differences were not significant.

The sensitivities during the follow-up periods were 51.7% (15/29) for AICS (gastric), 18.2% (2/11) for AICS (lung), 28.6% (8/28) for AICS (colorectal), 36.4% (8/22) for prostate, 29.0% (9/31) for AICS (breast), and 33.3% (2/6) for AICS (uterine/ovarian) (Fig. 2B, Table S1). The sensitivities of AICS were higher within 1 year than those at all follow-up periods, but the differences were not significant.

The sensitivities of AICS in this study compared to those in developmental case-control study^13^ were shown in Figs S2, S3. The sensitivity within 1 year after AICS examination was equal to or higher than that in a developmental case-control study.

### Positive predictive values

**Figure 3:**
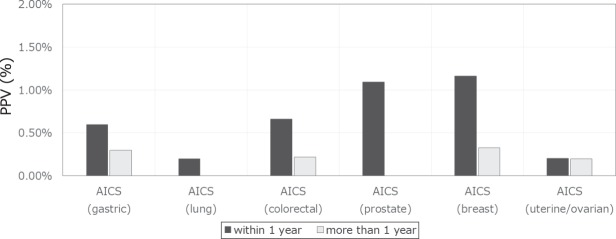
Positive predictive value of AICS for each type of cancer in rank C. The black and grey bars represent the positive predictive value within and after more than 1 year after AICS examination.

The positive predictive values (PPVs) in rank C within 1 year after AICS examination were 0.60% (10/1,660) for AICS (gastric), 0.20% (2/1,015) for AICS (lung), 0.66% (6/905) for AICS (colorectal), 1.09% (8/732) for AICS (prostate), 1.16% (7/606) for AICS (breast), and 0.20% (1/490) for AICS (uterine/ovarian). Meanwhile, the PPVs for cancer diagnosis at more than 1 year after AICS examination were 0.30% (5/1,650) for AICS (gastric), 0% (0/1,013) for AICS (lung), 0.22% (2/899) for AICS (colorectal), 0% (0/724) for AICS (prostate), 0.33% (2/599) for AICS (breast), and 0.20% (1/489) for AICS (uterine/ovarian) (Fig. 3, Table S2).

The PPVs of AICS in this study compared to those in developmental case-control study were shown in Figs S4, S5. PPVs within 1 year after AICS examination, those of AICS (gastric), AICS (lung), AICS (colorectal), and AICS (uterine/ovarian) were lower than those reported in a previous case-control study, while those of AICS (prostate) and AICS (breast) were higher.

**Figure 4:**
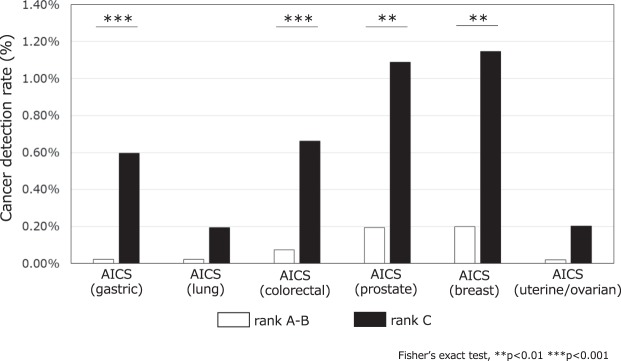
Cancer detection rate by each AICS rank. The white bars represent the cancer detection rate in AICS rank A and rank B, while the black bars indicate the rate in AICS rank C.

Meanwhile, the PPVs for cancer detection rates in rank A and rank B were 0.02% (2/8,585) for AICS (gastric), 0.02% (2/9,230) for AICS (lung), 0.07% (7/9,340) for AICS (colorectal), 0.20% (8/4,087) for AICS (prostate), 0.19% (9/4,820) for AICS (breast), and 0.02% (1/4,936) for AICS (uterine/ovarian) (Fig. 4). In AICS (gastric), AICS (colorectal), AICS (prostate), and AICS (breast), the cancer detection rate (PPVs) in rank C were significantly higher than those in rank A and B (AICS (gastric), P < 0.001; AICS (lung), P = 0.051; AICS (colorectal), P < 0.001; AICS (prostate), P = 0.001; AICS (breast), P = 0.001; and AICS (uterine/ovarian), P = 0.172).

## Discussion

In this study, we validated the usefulness of AICS as a cancer screening tool through a large multicentre prospective study of 10,245 subjects in Japan. We found that AICS is a more useful method for evaluating the probability of cancer incidence than for prediction of cancer onset.

Both the incidence and mortality rates of cancer are increasing worldwide^21^. Therefore, the implementation of methods for the prevention, early detection, and treatment of cancers is crucial to reduce mortality. Various screening methods have been established for cancers, but it can be expensive, time consuming, and physically and/or mentally taxing to undergo each screening examination separately. Blood cancer screening tests are useful modalities with a low physical burden, whereas most of the existing tumour markers are not adequate for early cancer detection^22^.

As previously reported, AICS was developed as a novel blood test for simultaneous screening of gastric, lung, colorectal, pancreatic, prostate, breast, and uterine/ovarian cancers based on the differences in PFAA in each cancer patient and healthy control^12–15^. These previous case-control studies reported that statistically significant changes in specific amino acid concentrations were observed even in the early stage of each cancer. Also, AICS has been reported to be a more useful tool for the early detection of various cancers compared to the existing tumour markers^23^.

In this study, the capability of AICS for cancer detection was prospectively investigated in various populations. AICS rank distribution was 56.3–71.4%, 19.7–28.5% and 8.8–16.2% for rank A, rank B and rankC, respectively. Sensitivities in rank C within 1 year after AICS examination were 83.3% 50.0%, 46.2%, 50.0%, 43.8%, and 50.0% for AICS (gastric), AICS (lung), AICS (colorectal), AICS (prostate), AICS (breast), and AICS (uterine/ovarian), respectively. The sensitivities and PPVs in rank C at more than 1 year were lower than those within 1 year. AICS was confirmed to be a more useful cancer screening tool for evaluating the probability of cancer incidence at the time of examination or within 1 year after AICS examination than for predicting cancer onset. Thus, AICS is recommended to be conducted annually for early detection of various cancers. Also, the results of AICS was suggested to reflect the cancer-bearing condition. We previously reported that AICS values decreased significantly after curative resection of various cancers^24–26^, suggesting that PFAA changes in AICS indicate cancer as a tumour marker.

Various possible mechanisms for PFAA profile changes in patients with cancer have been reported, including involvement of the metabolic changes in local cancer, induction of remote organ metabolic changes, or involvement of the immune system^27–29^. However, several points regarding the mechanisms behind changes in PFAA profiles in patients with cancer remain unclear, and thus further research is needed to clarify these issues.

In the current study, the sensitivity within 1 year after AICS examination was equal to or higher than that in a developmental case-control study^13^ (Figs S1, S2). The total cancer detection rate by AICS was 0.33% (34/10,245), which was superior to the 0.26% reported in the results of a national survey conducted by the Japan Society of Ningen Dock in 2015^30^.

Regarding PPVs within 1 year after AICS examination, those of AICS (gastric), AICS (lung), AICS (colorectal), and AICS (uterine/ovarian) were lower than those reported in a previous case-control study^13^, while those of AICS (prostate) and AICS (breast) were higher (Figs S3, S4).

In the previous case-control study, the PPV was calculated by estimating the morbidity according to nationwide age class in Japan (0.10% for gastric, 0.09% for lung, 0.13% for colorectal, 0.33% for prostate, 0.13% for breast, and 0.09% for uterine/ovarian cancer)^31^. In this study, the cancer incidence within 1 year after AICS examination was 0.12% for gastric, 0.04% for lung, 0.13% for colorectal, 0.33% for prostate, 0.29% for breast, and 0.04% for uterine/ovarian cancer. For accurate comparison of PPVs between different studies, the number of cancer incidence in each group should be considered.

Significantly higher cancer detection rates in rank C than those in rank A and B were observed, suggesting the usefulness of AICS to identify the population with the highest probability of cancer incidence, allowing for detailed examinations of each cancer.

In conclusion, AICS was validated to be a useful cancer screening tool to evaluate the risk of various cancer in a large scale multicentre prospective study. This indicates that annual AICS should be recommended to detect any malignancy.

## Methods

### Ethical considerations

This prospective observational study was performed in accordance of the Declaration of Helsinki, and the study’s protocol was approved by the ethics committees of Chiba Cancer Center, Mitsui Memorial Hospital, and Saihaku Hospital. All participants provided written informed consent for inclusion in the study. All data were analysed anonymously throughout the study.

### Subjects

The study enrolled individuals who underwent AICS in Chiba Cancer Center or Mitsui Memorial Hospital or Saihaku Hospital between January 2010 and December 2015.

### Measurement of plasma amino acid concentrations

Blood samples (5 mL) were collected from a forearm vein in the morning after an overnight fasting, using tubes containing ethylenediaminetetraacetic acid (Termo, Tokyo, Japan). These were immediately placed on ice. Plasma was prepared via centrifugation at 3,000 rpm (15 min at 4 °C) and was stored at −80 °C until the analysis. The plasma samples were deproteinised before the measurements using acetonitrile at a final concentration of 80%, and the PFAA concentrations were measured using high-performance liquid chromatography/electrospray ionization tandem mass spectrometry with pre-column derivatization. These analytical methods have been described previously^32–34^.

### Calculating AICS values and ranks

AICS values were classified as rank A, B, or C based on a previous report^13^. AICS values range from 0.0 to 10.0; a value of 5.0 and 8.0 has 80% and 95% specificity, respectively^13,17–20^. Higher values are associated with a greater probability of each cancer. For determining the probability of each cancer based on AICS value, the values were categorised into 3 as follows: rank A, 0.0–4.9; rank B, 5.0–7.9; and rank C, 8.0–10.0. In this study, the clinical performance of AICS (gastric), AICS(lung), AICS(colorectal), AICS(prostate), AICS(breast) and AICS(uterine/ovarian) were validated, while AICS (pancreatic) was excluded because because it was not commercially available.

### Sensitivity and positive predictive value

The sensitivity in rank C of each AICS was calculated as the ratio of the number of patients with cancer categorised as rank C to the number of patients in the overall cohort with the corresponding cancer type. The PPV in rank C was calculated as the ratio of the number of patients with cancer categorised as rank C to the total cohort categorised as rank C. At the Chiba Cancer Center, cancer incidence was calculated from collated data from the regional cancer registry (death certificate notification: 8.8%; death certificate only: 3.4%). In Mitsui Memorial Hospital and Saihaku Hospital, detailed examinations were performed for rank C subjects, and information on cancer incidence was collected from the results of these detailed examinations. Also, subjects in Saihaku Hospital were tracked based on regional follow-up surveillance. Information on cancer incidence in rank A and rank B subjects was collected from the results in the health check-up records.

### Statistical analysis

All statistical analyses were performed using Fisher’s exact test. All analyses were performed using R software (version 3.2.2; The R Foundation for Statistical Computing, Vienna, Austria). P < 0.05 was considered significant.

## Supplementary information

Supplementary Infromation

## Data Availability

Requests for data and materials should be addressed to the corresponding author.
