# Supplementary material for: A multicentre clinical validation of AminoIndex Cancer Screening (AICS)
Source: Sci Rep. 2019 Sep 25;9:13831. doi: 10.1038/s41598-019-50304-y (PMC6761170; doi:10.1038/s41598-019-50304-y)
Supplement: Supplementary file 1 — Supplementary Infromation [file 41598_2019_50304_MOESM1_ESM.pdf]

Supplementary Information

A multicentre clinical validation of the AminoIndex Cancer Screening (AICS)

Haruo Mikami[1*], Osamu Kimura[2], Hiroshi Yamamoto[3], Shinya Kikuchi[3], Yohko Nakamura[1], Toshihiko Ando[3], Minoru Yamakado[4,5]

[1]Chiba Cancer Center Research Institute, Chiba, Japan

[2]Hakuai Hospital, Tottori, Japan

[3]Ajinomoto Co. Inc., Kanagawa, Japan

[4]Department of Nursing, Ashikaga University, Tochigi, Japan

[5]Center for Multiphasic Health Testing and Services, Mitsui Memorial Hospital, Tokyo, Japan

Supplementary figure legends

Figure S1. AICS rank distribution of each cancer patient within 1 year after AICS examination
White, grey, and black parts show the distribution of rank A, B, and C, respectively.

Figure S2. Sensitivity of AICS within 1 year compared to development data
The white and black bars represent the sensitivities in development data and within 1 year after AICS examination, respectively. Significant difference between groups ($p<0.05$, Fisher's exact test).

Figure S3. Sensitivity of AICS through all follow-up periods compared to development data
The white and grey bars represent the sensitivities in development data and through all follow-up periods, respectively.

Figure S4. Positive predictive value of AICS within 1 year compared to development data
The white and black bars represent the positive predictive values in development data and within 1 year after AICS examination, respectively.

Figure S5. Positive predictive value of AICS through all follow-up periods compared to development data
The white and grey bars represent the positive predictive values in development data and through all follow-up periods, respectively.

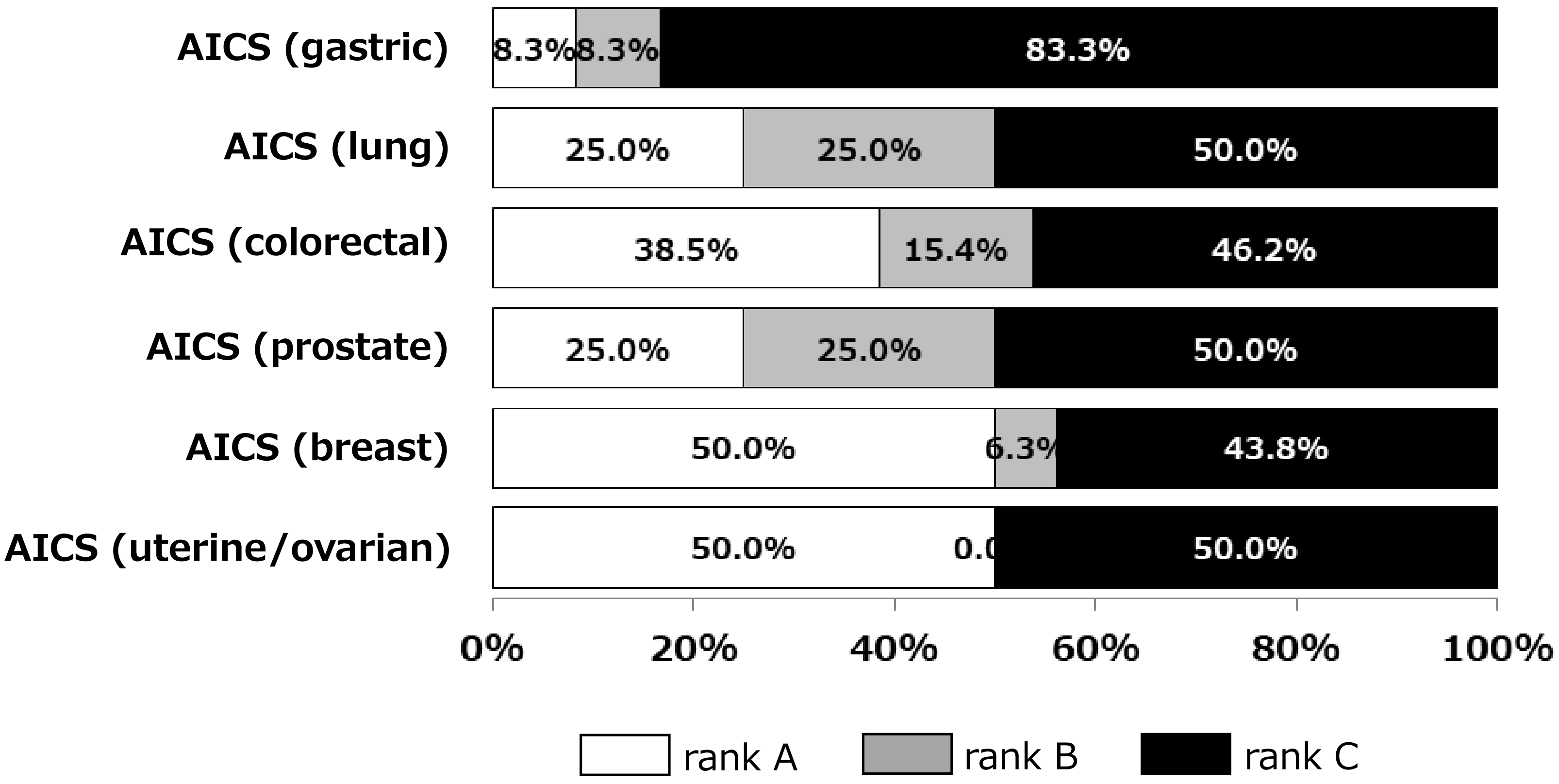

AICS (gastric)
8.3%
8.3%
83.3%
AICS (lung)
25.0%
25.0%
50.0%
AICS (colorectal)
38.5%
15.4%
46.2%
AICS (prostate)
25.0%
25.0%
50.0%
AICS (breast)
50.0%
6.3%
43.8%
AICS (uterine/ovarian)
50.0%
50.0%
0%
20%
40%
60%
80%
100%
rank A
rank B
rank C

Figure S1

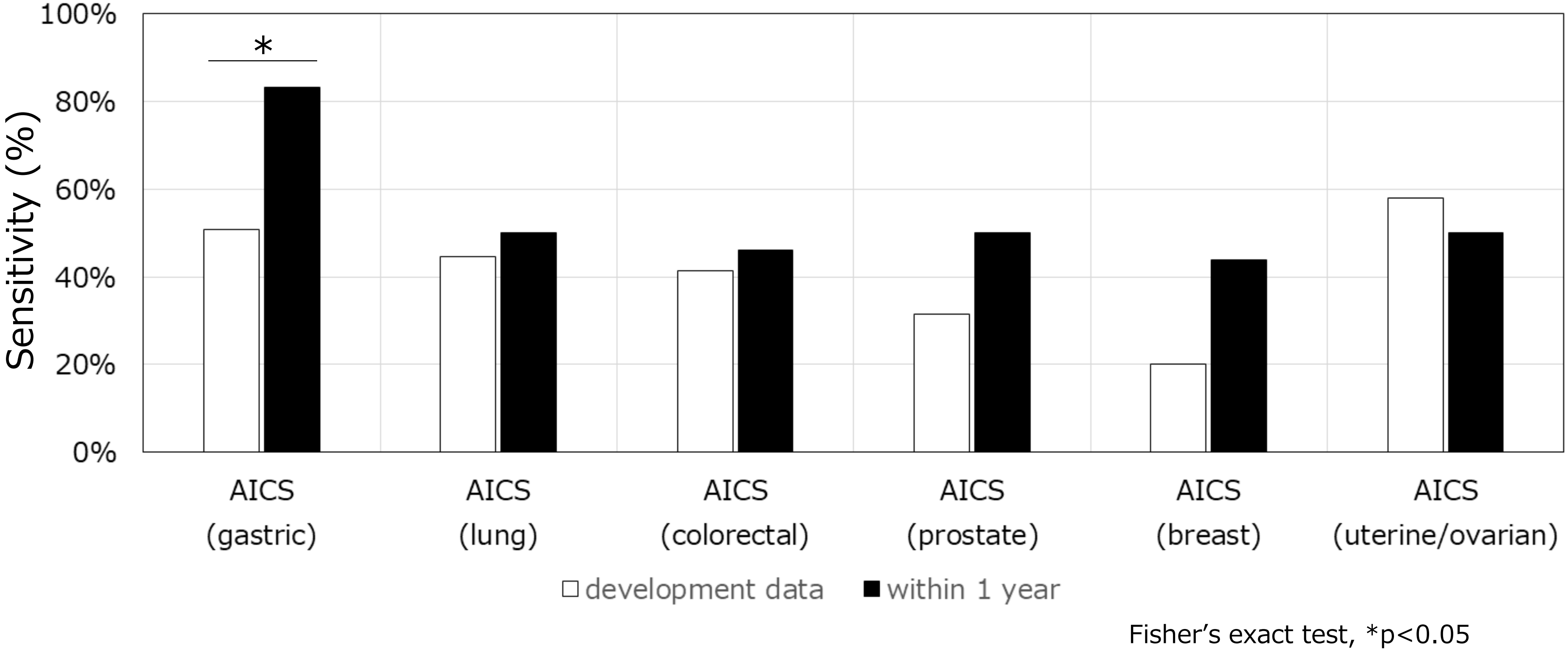

sensitivity	AICS (gastric)	AICS (lung)	AICS (colorectal)	AICS (prostate)	AICS (breast)	AICS (uterine/ovarian)
Development data	50.8%	44.6%	41.4%	31.5%	20.0%	58.1%
within 1 year	83.3% (10/12)	50.0% (2/4)	46.2% (6/13)	50.0% (8/16)	43.8% (7/16)	50.0% (1/2)

Figure S2

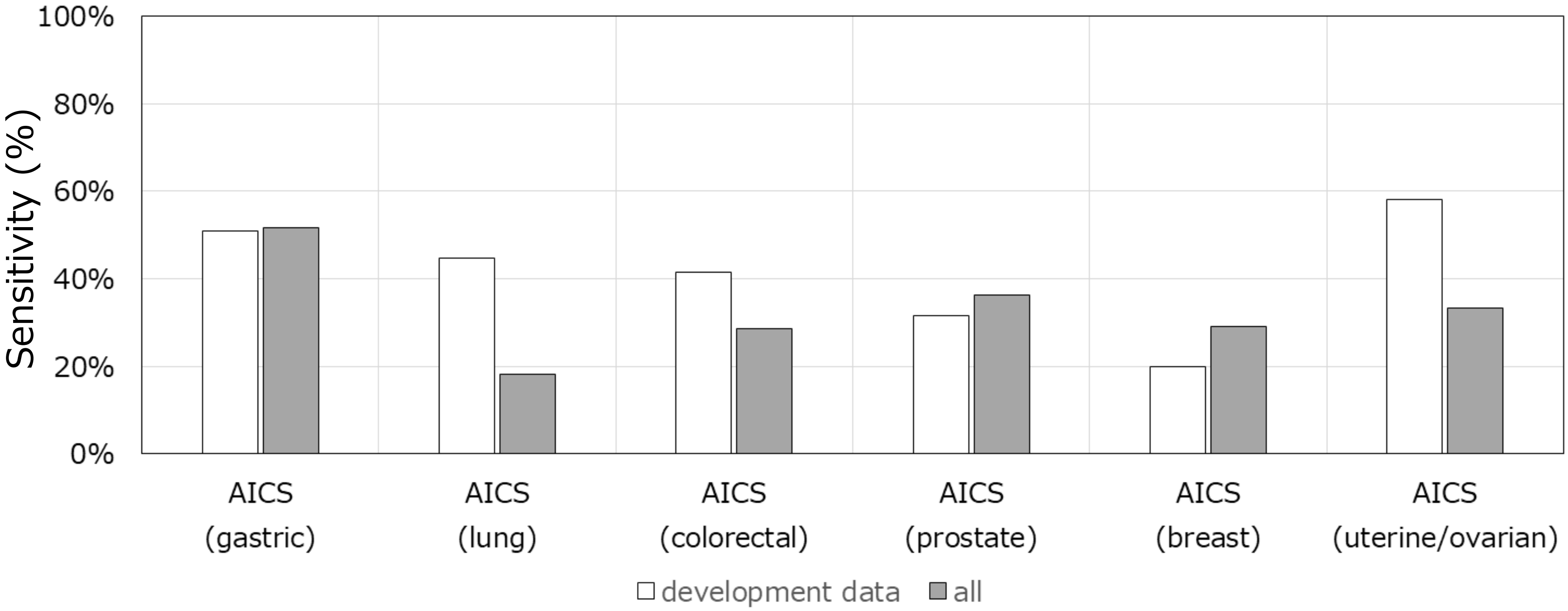

sensitivity	AICS (gastric)	AICS (lung)	AICS (colorectal)	AICS (prostate)	AICS (breast)	AICS (uterine/ovarian)
Development data	50.8%	44.6%	41.4%	31.5%	20.0%	58.1%
Maximum follow-up of 6.2 years	51.7% (15/29)	18.2% (2/11)	29.2% (8/28)	36.4% (8/22)	29.0% (9/31)	33.3% (2/6)

Figure S3

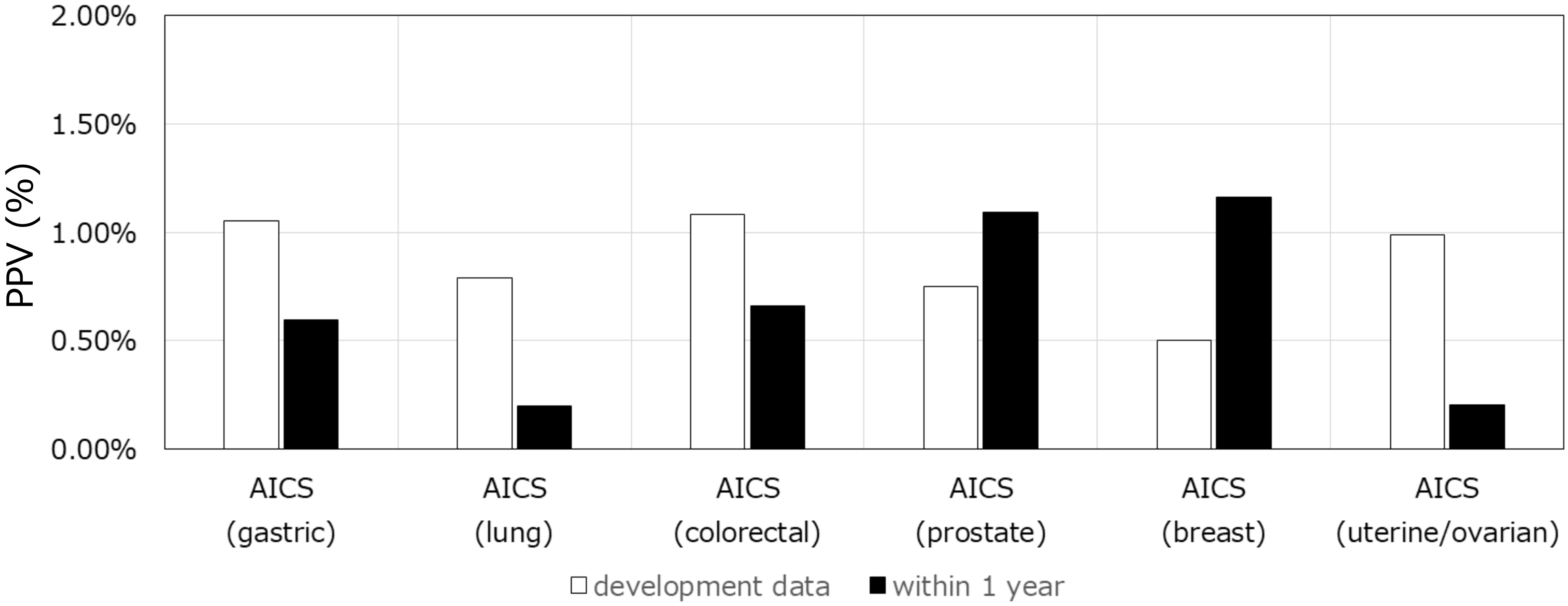

PPV	AICS (gastric)	AICS (lung)	AICS (colorectal)	AICS (prostate)	AICS (breast)	AICS (uterine/ovarian)
Development data	1.05%	0.79%	1.08%	0.75%	0.50%	0.99%
within 1 year	0.60% (10/1660)	0.20% (2/1015)	0.66% (6/905)	1.09% (8/732)	1.16% (7/606)	0.20% (1/490)

Figure S4

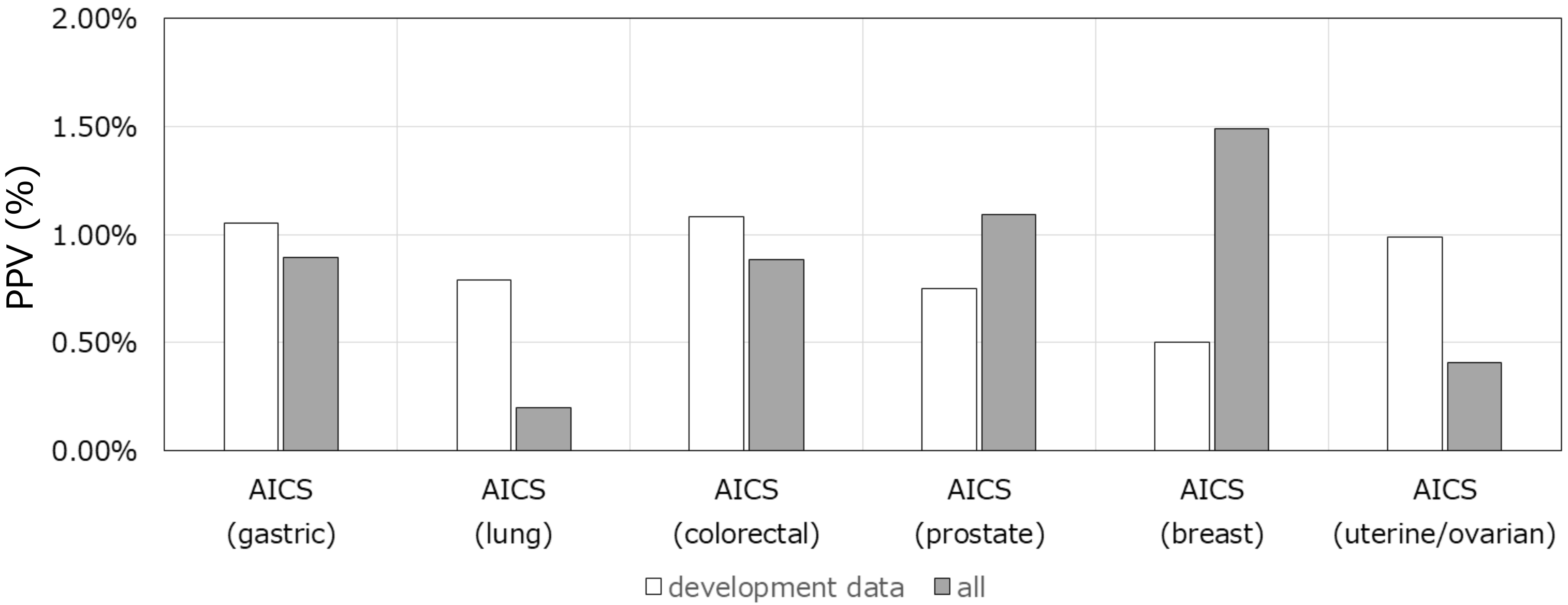

PPV	AICS (gastric)	AICS (lung)	AICS (colorectal)	AICS (prostate)	AICS (breast)	AICS (uterine/ovarian)
Development data	1.05%	0.79%	1.08%	0.75%	0.50%	0.99%
Maximum follow-up of 6.2 years	0.90% (15/1660)	0.20% (2/1015)	0.88% (8/905)	1.09% (8/732)	1.49% (9/606)	0.41% (2/490)

Figure S5

Table S1. Sensitivity of AICS compared to that of development data

Sensitivity	AICS (gastric)	AICS (lung)	AICS (colorectal)	AICS (prostate)	AICS (breast)	AICS (uterine/ovarian)
Within 1 year	83.3% (10/12)	50.0% (2/4)	46.2% (6/13)	50.0% (8/16)	43.8% (7/16)	50.0% (1/2)
More than 1 year	29.4% (5/17)	0% (0/7)	13.3% (2/15)	0% (0/6)	13.3% (2/15)	25.0% (1/4)
Maximum follow-up of 6.2 years	51.7% (15/29)	18.2% (2/11)	28.6% (8/28)	36.4% (8/22)	29.0% (9/31)	33.3% (2/6)
(Development data[1)])	(50.8%)	(44.6%)	(41.4%)	(31.5%)	(20.0%)	(58.1%)

1) Okamoto N, Use of “AminoIndex technology” for cancer screening. Ningen Dock. 2012;26:911-922.

Table S2. Positive predictive value of AICS compared to that of development data

PPV	AICS (gastric)	AICS (lung)	AICS (colorectal)	AICS (prostate)	AICS (breast)	AICS (uterine/ovarian)
Within 1 year	0.60% (10/1660)	0.20% (2/1015)	0.66% (6/905)	1.09% (8/732)	1.16% (7/606)	0.20% (1/490)
More than 1 year	0.30% (5/1650)	0% (0/1013)	0.22% (2/899)	0% (0/724)	0.33% (2/599)	0.20% (1/489)
Maximum follow-up of 6.2 years	0.90% (15/1660)	0.20% (2/1015)	0.88% (8/905)	1.09% (8/732)	1.49% (9/606)	0.41% (2/490)
(Development data[1)])	(1.05%)	(0.79%)	(1.08%)	(0.75%)	(0.50%)	(0.99%)

1) Okamoto N, Use of “AminoIndex technology” for cancer screening. Ningen Dock. 2012;26:911-922.
